# Association between cardiovascular manifestation and COVID‐19

**DOI:** 10.1002/clc.23423

**Published:** 2020-07-15

**Authors:** Kosuke Miki, Teruhiko Imamura

**Affiliations:** ^1^ University of Toyama Toyama Japan; ^2^ Second Department of Internal Medicine University of Toyama Toyama Japan

To the editor,

Cardiovascular manifestation is receiving great concern as both cause and result of coronavirus disease 2019 (COVID‐19).^1^ Chen et al systemically observed the association between COVID‐19 and cardiovascular status including myocardial injury, blood pressure, and arrhythmia.^2^ Several concerns that should improve their findings are raised.

More detailed examinations would clarify the involvement of cardiovascular system.^3^ The echocardiographic methodology of how to assess left and right heart failure is unclear in their study. Quantification of cardiac function including strain assessment and tissue Doppler analyses would be used. Measurement of QRS duration would let us assess the electrocardiographic myocardial injury. Image study to survey coronary artery disease would also be required.

The second concern is a time course of COVID‐19 in their study. Some patients might have progressed from severe status to critical status, and others might have died. In addition to the prediction of critical status, readers might have an interest in predicting mortality using baseline characteristics.

The third concern is an intervention to the cardiovascular system. They showed that myocardial injury was an independent risk factor of critical status. Aggressive treatment using anti‐heart failure agents including beta‐blocker might prevent the progression of COVID‐19.
